# Lattice Strain Due to an Atomic Vacancy

**DOI:** 10.3390/ijms10062798

**Published:** 2009-06-19

**Authors:** Shidong Li, Michael S. Sellers, Cemal Basaran, Andrew J. Schultz, David A. Kofke

**Affiliations:** 1 Electronic Packaging Laboratory, University at Buffalo, The State University of New York 14260-4300, USA; E-Mail: cjb@eng.buffalo.edu (C.B.); 2 Department of Chemical Engineering, University at Buffalo, The State University of New York, USA; E-Mails: msellers@buffalo.edu (M.S.S.); ajs42@buffalo.edu (A.S.); dkofke@buffalo.edu (D.K.)

**Keywords:** lattice strain, virial stress, vacancy transport, electromigration, thermomigration, embedded-atom method, molecular dynamic simulations

## Abstract

Volumetric strain can be divided into two parts: strain due to bond distance change and strain due to vacancy sources and sinks. In this paper, efforts are focused on studying the atomic lattice strain due to a vacancy in an FCC metal lattice with molecular dynamics simulation (MDS). The result has been compared with that from a continuum mechanics method. It is shown that using a continuum mechanics approach yields constitutive results similar to the ones obtained based purely on molecular dynamics considerations.

## 1. Introduction

In a work published in 1976, Blech [1] showed that the atomic vacancy flux process creates a stress gradient during electromigration. When this stress gradient is large enough, the electromigration process cannot happen in metals if the cathode and anode are within a certain maximum distance. This stress-vacancy relationship is referred to as *Blech’s critical length*. In 1993, Kirchheim [2] proposed a model that reached to the microscopic level, describing the generation of tensile and compressive stresses in aluminum lines. He used the instances of atomic vacancy generation, annihilation, and transport to account for these electromigration induced stresses. In 1999, Gleixner and Nix [3] proposed another model for electromigration and stress-induced void formation in aluminum VLSI interconnects based on classical nucleation theory. In that work they provide a discussion of an upper limit for hydrostatic tensile stresses in such lines based on an assumed volumetric lattice strain value. Kirchheim's model has been expanded by Sarychev*, et al*. [4] and Bassman [5]. Sarychev *et al*. state that the main disadvantage of Kirchheim's approach is its neglect of vacancy flux in the stress evolution of the system. Their model offers a method for connecting the evolution of the stress tensor with the transport of vacancies, the geometry of the metallization, and the stress and displacement boundary conditions that apply to it. In a dissertation by Bassman, a thermodynamic formalism for both the vacancy contribution to stress and chemical potential gradients was developed. Her work investigates stress-mediated self-diffusion in polycrystalline solids. In all three models, the size of an aluminum atomic vacancy is characterized by the strain that the volume of an atom would undergo upon its removal from a perfect lattice.

The change in volume describing a vacancy is related to the original atomic volume by the parameter *f,* called the vacancy relaxation factor. The form of Sarychev’s relation is shown below in equation (1):

(1) $$\varepsilon_{v}=-f\Omega <0$$

where ε*_v_* is the strain deformation introduced by vacancy volume relaxation, *f* is the vacancy relaxation factor which is a dimensionless number, and *Ω* is the volume of an atom. However, apparent dimensional inconsistency is observed in equation (1). Sarechev’s notation will be abandoned in our context, which will be more reasonably described by Kirchheim’s form which defined *f* as the volumetric strain induced by replacing a matrix atom with a vacancy. Following Kirchheim’s definition, the volume change induced by generation/annihilation of a vacancy can be expressed by:

(2) $$V_{f}=(1-f)\Omega$$

Equation (2) assumes that vacancy behaves like a foreign atom with smaller volume, *(1-f)Ω,* than that of a matrix atom. Very early analytical work investigating the value of *f* was conducted by Doyama and Cotterill [6]. Their work calculated the volume of a vacancy by computing the change in positions of copper atoms in a crystal. A pairwise Morse potential described atomic interactions nearest to the point defect, treating the atoms as discrete particles. Further away, atoms were susceptible to treatment by the elastic theory. Doyama and Cotterill found the volume of a copper vacancy to be 0.83*Ω, where *Ω* is the atomic volume. Gleixner and Nix, in their work mentioned earlier, and Shawman [7], report that for FCC metals, the vacancy volume is 0.9*Ω.

Our work attempts to provide a more accurate representation of this relaxation factor, both in atomic scale and in continuum mechanics scale. By comparing results from methods applicable in different length domains, constitutive values are sought for multiscale material modeling.

## 2. Molecular Dynamics Simulation Details

### 2.1. Embedded-Atom Method

The interactions between aluminum atoms in our simulation are characterized by Daw and Baskes' embedded-atom method [8,9]. This method serves as a desirable alternative to simpler, pair-wise approaches because of the EAM's realistic description of metallic cohesion and is discussed in a comprehensive review [10] and detailed in other papers [11,12]. A brief summary of the method, based on these works, is presented here.

Daw, Foiles, and Baskes [10] proposed that the major contribution to the energetics of a metal is the energy to embed an atom into the electron density of neighboring atoms. The remaining energy is explained by a short-range, doubly screened pair interaction that accounts for core-core repulsions. Thus, the total energy of the system is written as:

(3) $$E_{tot}=\sum_{i}F_{i}(\rho_{h,i})+\frac{1}{2}\sum_{i,j(i\neq j)}\phi_{ij}(R_{ij})$$

Here, *F_i_* is the embedding energy for placing an atom in a host electron density. That density is described by *ρ_h,i_* which is the total electron density at atom *i*, due to the rest of the atoms in the system. We can simplify the description of *ρ_h_*_,_*_i_* by assuming that the host density is closely approximated by a sum of the atomic densities, $\rho_{j}^{a}$ of the neighbors *j* of atom *i*:

(4) $$\rho_{h,i}=\sum_{j,\neq i}\rho_{j}^{a}(R_{ij})$$

These atomic densities are, as shown in equation (4), merely functions of position and provide straightforward calculation of the embedding energy of the atom in question.

The embedding function, *F_i_*, maintains its simplicity when calculating an atom in an alloy versus a pure material, as it does not depend on the source of the electron density, but only on atom *i*. The second term in equation (3), *φ_ij_* represents the pair interaction and is purely repulsive. Both the embedding function and pair interaction terms are derived on a per material basis, calculated from the formal definitions within the author's density-functional framework, as well as fitting them to describe the bulk equilibrium solid's properties—specifically, the equilibrium lattice constant, heat of sublimation, elastic constants, vacancy formation energy, and BCC-FCC energy difference. The specific potential file for aluminum used with the EAM was developed by Mishin and Farkas, *et al*. in 1999. Compared to other aluminum potential files for EAM, this accurately reproduces basic equilibrium properties of aluminum derived from both *ab initio* and experimental data, as well as the correct relative stability of different alternative structures with coordination numbers ranging from 12 to 4. This latter feature is particularly desirable for this study.

### 2.2. Virial Stress

The virial definition of atomic stress is used to calculate the stress around a given volume of simulation space:

(5) $$\sigma_{\alpha ,\beta }=-\frac{1}{V}\left[\frac{1}{2}\sum_{i}\sum_{j,\neq i}F_{i,j}^{\beta }(r_{i}^{\alpha }-r_{i}^{\alpha })+\sum_{i}mv^{\alpha }v^{\beta }\right]$$

Here, F_ij_ is the force on an atom *i* by atom *j* in the β direction, multiplied by the components of the distance between *i* and *j* in the α direction. The second term represents the kinetic portion of the internal pressure of the system. Where *m* is the mass of atom *i*, and *v* is the particular component of its velocity in directions α and β. V is the volume containing the atoms *i* and *j* included in the equation.

There have been many researchers who have questioned point-wise stress calculation in a system using the expression for atomic stress taken from the virial theorem. Zimmerman *et al*. [13] show that an expression for continuum mechanical stress in atomistic systems, derived by Hardy [14] converges quicker than the viral to values expected from continuum theory, as a function of volume. Zhou [15] argues that neither the virial stress, which includes total atomic velocities, nor Hardy's stress, which includes velocity fluctuations, represent a measure of the true mechanical stress. We present here our results calculated from the virial form of the stress, as this was more convenient to implement. However, the points made in the preceding works will be considered during the continuation of this research.

### 2.3. Molecular Dynamics Simulations

For the aluminum simulations, the LAMMPS molecular dynamics simulation software package was used [16]. Data collection runs were conducted using a constant number of particles, constant volume, and at a constant temperature (NVT) for pure aluminum in an FCC lattice at 533K. These conditions are the same as in Sarychev’s work (see Table 1). A simulation box size of 6 x 6 x 6 lattice lengths with periodic boundary conditions was used and an initial FCC lattice unit cell length was set at 4.032 angstroms. The system was first equilibrated with an NPT style integrator to allow the lattice (volume) to expand to its zero pressure value at 533K. Next, the system was switched to an NVT integrator for data collection. Simulations were controlled with a Nose-Hoover thermostat and integrated with time steps of 0.001 picoseconds.

The system was first allowed to converge to equilibrium, which we simulated for 40 picoseconds (ps). Following achieving a convergence, atomic positions and point-wise stresses were collected every 0.5ps for duration of 10.0 ps under NVT conditions. Next, a void was created by removing an atom from the lattice. Data collection continued for 10.0 ps. Atom specific positions and stresses were collected for the original atom's 12 first nearest-neighbors before and after atom removal.

## 3. Lattice Volumetric Strain

The volumetric strain created after the removal of an atom is found by direct measurement of first-nearest neighbor positions. Prior to the vacancy formation, distances between each first-nearest neighbor and the atom to be removed were recorded every 0.5ps (500 time steps), for 10.0ps (10^3^ time steps). Averaging the first-nearest neighbor positions, we can find the center of the void, and from there an average neighbor distance from the void, R_1_, is found. Next, the atom is removed and the system is allowed to converge to an equilibrium which took about 500 time steps. Similar to before, distances between each first-nearest neighbor and the center of the void are recorded every 500 time steps, for 10^3^ time steps. A second average neighbor distance, R_2_, is found. Spherical volumes based on these two radii are computed and the volumetric strain is computed as shown below.

(6) $$\varepsilon_{v}=\frac{\Omega_{1}-\Omega_{2}}{\Omega_{1}}=\frac{{\left(R_{1}\right)}^{3}-{\left(R_{2}\right)}^{3}}{{\left(R_{1}\right)}^{3}}$$

**Figure 1.**
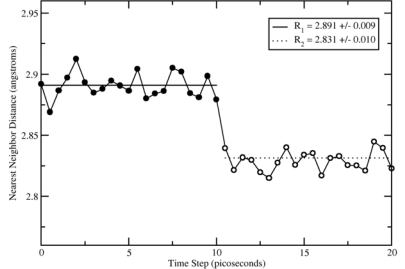
A plot of first-nearest neighbor distance from center of an atom (or void), versus simulation time steps in molecular dynamic simulations. Filled black circles indicated a full lattice and open circles indicate a vacancy, where the atom is removed at 10 ps into the data collection run. Average neighbor positions before and after atom removal are 2.891 +/–0.009 and 2.831 +/–0.010, respectively.

The average initial (R1) and final (R2) distances to the atom or void center for one particular molecular dynamics run are shown in Figure 1. Dotted lines of (R1) and (R2) values are averages over multiple simulation runs, with statistical uncertainty shown in the legend. From our first-nearest neighbor distance, and using *equation* (6), we obtain that *f*_I_ = 0.060 +/–0.013. The error found here is based on uncertainty in (R1) and (R2), propagated through *equation* (6). The values reported by authors doing similar research are listed in Table 2.

## 4. Validation with Continuum Mechanics Methods

**Figure 2.**
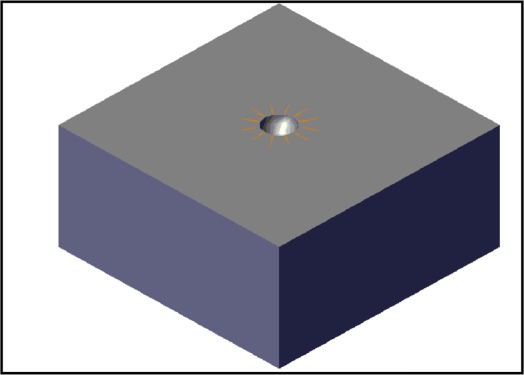
Void model in continuum mechanics domain.

In this section, continuum mechanics formulations are introduced to calculate the spherical stress induced by the removal of a matrix atom. The location of the missing atom is simplified as a spherical cavity inside an infinite elastic body. The interactions between atoms, including short range repulsion and long distance attraction, are the source of the stresses in continuum level. After the sudden removal of an atom, the attraction can no longer be balanced by the repulsion. Hence the atoms nearby will sink into the void until they reach another balance. This is the mechanism of shrinkage strain at the missing atom site. In this method, the atoms interactions are treated as hydrostatic pressure around the cavity. By introducing the elastic constitutive relationship, the volumetric stress can be calculated, which is shown in Figure 2.

(7) $$P=\frac{1}{3}\left(\sigma_{r}+\sigma_{\theta }+\sigma_{\phi }\right)$$

**Figure 3.**
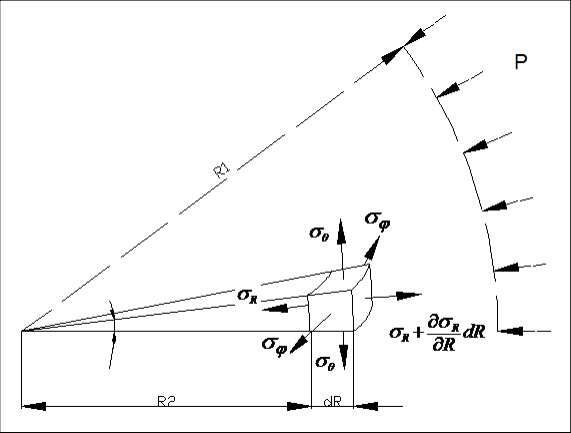
Free body diagram under spherical coordinate system.

Shown in Figure 3 is a free body diagram of our sphere under stress. Further analysis of these stresses show that at the inner boundary of the cavity, we have:

(8) $$\sigma_{r}=0$$

(9) $$\sigma_{\theta }=\sigma_{\phi }=\frac{3}{2}P$$

By Hooke's Law in spherical coordinates, we have:

(10) $$\varepsilon_{\theta }=-\frac{v\sigma_{r}}{E}+\frac{(1-v)\sigma_{\theta }}{E}$$

where *E* is Young's modulus and *ν* is Poisson's ratio. Volumetric strain from the strain of our sphere in the θ direction can be obtained by:

(11) $$\varepsilon_{v}=\varepsilon_{\theta }+\varepsilon_{r}+\varepsilon_{\phi }$$

Applying equations (7) to (11), the spherical stress is calculated to be −2029.52 MPa, which is about 32% smaller than the virial stress P = −2978.8 MPa. As stated in the previous section, in continuum mechanics, stress is defined as the internal force intensity across an imaginary face. It doesn’t consider the particles cross over the boundary. While in molecular dynamics, virial stress measures the momentum change in definite group of particles. Only when the density change is negligible can virial stress be approximated to be Cauchy stress. In this example, a reduction factor between atomistic and macroscopic scale will be needed to precisely consider the bulk modulus difference in between atomic scale method and continuum mechanics method. However, considering the small density of vacancies in the total lattice sites, *C_v_/C_a_≈1×10^–5^* [17], the vacancy relaxation factor can be safely estimated to be 0.06~0.10 in most cases.

## 5. Components of Bulk Modulus

According to Blech’s relationship [1], the vacancy density in a system depends on its stress level. Therefore, by simply changing the stress status, we can study the role of vacancy sink/source in the constitutive relations. The equilibrium vacancy concentration with the following form can be found by simple manipulation on Blech’s relationship:

(12) $$c_{ve}=c_{v0}\text{exp}\left(\frac{(1-f)\Omega \sigma_{sp}}{kT}\right)$$

The vacancy generation/annihilation has the following rate dependent form:

(13) $$G=-\frac{c-c_{ve}}{\tau_{s}}$$

where τ*_s_* is vacancy relaxation period. It is a material property representing the period that the system needs to reach vacancy equilibrium, which is in the magnitude of 1*ms*.

**Figure 4.**
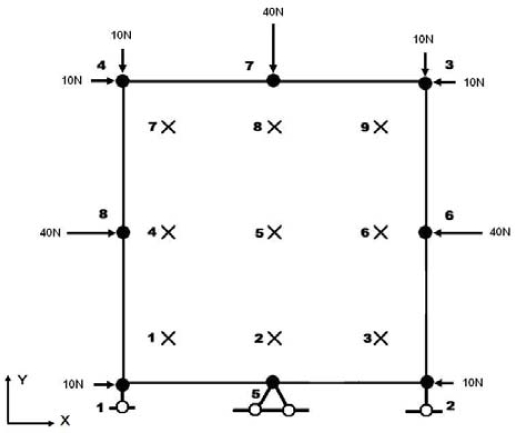
Plane strain element under compressive load.

From equation (12) we can see that tensile stress yields more vacancies while compressive stress results in fewer vacancies in metal. This model is employed as a user-defined element in ABAQUS. An 8 nodes plane strain element with the size of 2 mm×2 mm is stressed by the nodal forces and constraints as is shown in Figure 4. By simple manipulation, the stress state can be found to be *σ_x_=σ_y_=–*30 MPa and *τ_xy_=0*. In case of Poisson’ ratio ν = 0.33 and Young’s Modulus E = 62 GPa, considering no vacancies annihilation induced strain, *σ_z_* can be calculated to be *–19.8 MPa*, strain *ε_x_=ε_y_ =–*2.19×10^–4^, spherical stress *σ^spherical^ =–*26.6MPa, and volumetric strain *ε_V_ =–*4.38×10^–4^.

By taking the vacancies annihilation induced strain into consideration, the stress/strain state is calculated to be *σ_z_=* −19.5 MPa, *ε_x_=ε_y_ =–*2.25×10^–4^, *σ^spherical^=* −26.5 MPa and *ε_V_=*–4.50×10^–4^. The contraction due to vacancies annihilation reduces the compressive stresses in Z direction (normal to the plane). As a plane strain element, it is assumed strain in the direction normal to the plane is zero. In other words, there are constraints to prevent the deformation in Z direction. When the density of lattice sites decrease under compressive stress, the element tends to contract in all directions and thus reduces the reaction forces in Z direction.

By changing the sign of the pressure applied, it is found that the model with vacancies generation/annihilation mechanism yields more tensile strain than the one without does. Therefore, it can be concluded that the bulk modulus κ is composed by two parts:

(14) $$\kappa =\kappa_{e}+\kappa_{v}$$

where κ_e_ is due to the interaction between atoms. Tensile strain increases the bond distances between atoms and yields an overall tensile stress; compressive strain shortens the bond distances and results in compressive stress.

κ_v_ reflects the change due to the vacancy concentration. Tensile stress exaggerates the grain boundaries by producing more vacancies; compressive stress tends to merge the grain boundaries and thus reduces vacancies. Both generation and annihilation of vacancies result in the corresponding volumetric strain. κ_v_ can be derived from equation (14). It is usually a negative value, which depends on stress and load rate.

In case of loading time is much larger than vacancy relaxation time, as usually is, κ_v_ is much smaller than κ_e_. In this example, κ=59 GPa, κ_e_=60.7 GPa, and κ_v_=–1.75 GPa. κ_v_ is only 2.7% of κ_e,_ which makes it reasonable to approximate κ*_e_* ≈ κ since most available experimental data are bulk modulus upon its specific load rate and stress.

## 6. Conclusions

Using LAMMPS molecular dynamics simulator with the embedded-atom method, we simulated an aluminum lattice at 533K. We outputted atom positions and virial stresses for a particular atom and its first-nearest neighbors. That particular atom was then removed, and we used the change in positions to calculate the volume strain due to the creation of a void. We also calculated the volumetric strain induced spherical stress with continuum mechanics constitutive. The comparison of mechanical stress and virial stress shows that reduction factor is needed in order to bridge material modeling methods applicable in atomic scale to macroscale.

We also report that bulk modulus can be divided into two parts: one due to atomic bonds length and the other induced by vacancy sinks and sources mechanism. The latter part is strain rate dependent which can be negligible at static load.

## Figures and Tables

**Table 1.** Al material properties used in simulations.

**E**	Young's modulus, (111) texture	6.6x10^4^ MPa at (533K)
**υ**	Poisson’s Ratio	0.3496
**Ω**	Volume per Al atom, bulk	1.38x10^–23^ cm^3^

**Table 2.** Vacancy relaxation factors as reported by authors.

**Sarychev, *et al*.**	0.60
**Bassman**	0.20
**Doyama, *et al*.**	0.17
**Gleixner and Nix**	0.10
**This work**	0.060
